# Transoral robotic surgery for human papillomavirus-positive oropharyngeal squamous cell carcinoma: a retrospective observational study from a UK tertiary centre

**DOI:** 10.1017/S0022215126104599

**Published:** 2026-06

**Authors:** Rebecca Yerin Im, Conrad Orori, Jonathan Hughes, Paul Stimpson

**Affiliations:** 1 UCL Medical School, University College London, London, UK; 2 Head and Neck Surgery ENT, University College London Hospitals NHS Foundation Trust, London, UK

**Keywords:** carcinoma, squamous cell, oropharyngeal neoplasms, human papillomavirus 16, neoplasm staging, robotic surgical procedures

## Abstract

**Background:**

Human papillomavirus (HPV)-positive oropharyngeal squamous cell carcinoma (SCC) has favourable oncological outcomes, supporting the use of transoral robotic surgery with risk-adapted adjuvant therapy. While anticipated adjuvant treatment intensity is informed by pre-operative assessment, definitive post-operative histopathology determines final risk stratification.

**Methods:**

A single-centre study was conducted at University College London Hospitals of 44 HPV-positive oropharyngeal SCC patients treated with primary transoral robotic surgery and selective neck dissection. Discrepancies between clinical and pathological staging, and their impact on adjuvant treatment recommendations, were assessed.

**Results:**

Staging discrepancies were identified in 38 per cent of patients. Pathological nodal upstaging occurred in 20.5 per cent of patients, commonly caused by higher-than-anticipated nodal burden. Involved surgical margins were present in 70.5 per cent of patients. Overall, definitive histopathology resulted in escalation of anticipated adjuvant treatment intensity in 61 per cent of patients, with 52 per cent reclassified as high-risk disease.

**Conclusion:**

Post-operative histopathology frequently altered anticipated risk stratification, highlighting the limitations of pre-operative staging and the clinical impact of a surgical-first approach.

## Introduction

Oropharyngeal squamous cell carcinoma (SCC) can arise from the tonsils, base and posterior one-third of the tongue, soft palate or lateral pharyngeal walls. While historically associated with tobacco and alcohol exposure,^1^^,^^2^ UK data suggest human papillomavirus (HPV) is attributable in approximately half of oropharyngeal SCC, with HPV-16 predominant.^3^^,^^4^ HPV-positive disease is associated with a significantly improved prognosis compared with HPV-negative disease.^5^^,^^6^ Patients with HPV-positive oropharyngeal SCC are typically younger, have favourable performance status and have small primary tumours, although often presenting with disproportionate nodal disease.^6^^,^^7^ Locally advanced disease is managed with curative intent using either primary surgery with adjuvant (chemo)radiotherapy or primary (chemo)radiotherapy.

Given the favourable outcomes associated with HPV-positive oropharyngeal SCC, contemporary treatment strategies increasingly focus on minimising long-term treatment-related morbidity, without compromising disease control. Surgical-first approaches using transoral robotic surgery with selective neck dissection enable definitive pathological assessment and post-operative risk stratification, allowing adjuvant treatment to be tailored according to pathological findings rather than pre-operative staging alone.

In routine practice, discrepancies between pre-operative clinical staging and post-operative pathological staging may alter post-operative risk classification and adjuvant therapy recommendations. We evaluated staging discrepancies in a consecutive cohort of HPV-positive oropharyngeal SCC patients treated with primary transoral robotic surgery and selective neck dissection at University College London Hospitals and assessed the implications for post-operative adjuvant treatment within a tertiary referral service.

## Materials and methods

### Study design and setting

This was a single-centre retrospective observational study of patients with HPV-positive oropharyngeal SCC who underwent primary transoral robotic surgery with selective neck dissection at University College London Hospitals between 26 November 2020 and 7 November 2024. All work was conducted as part of institutional clinical evaluation within University College London Hospitals and student research within University College London medical school.

### Patient identification and eligibility

**Figure 1.**
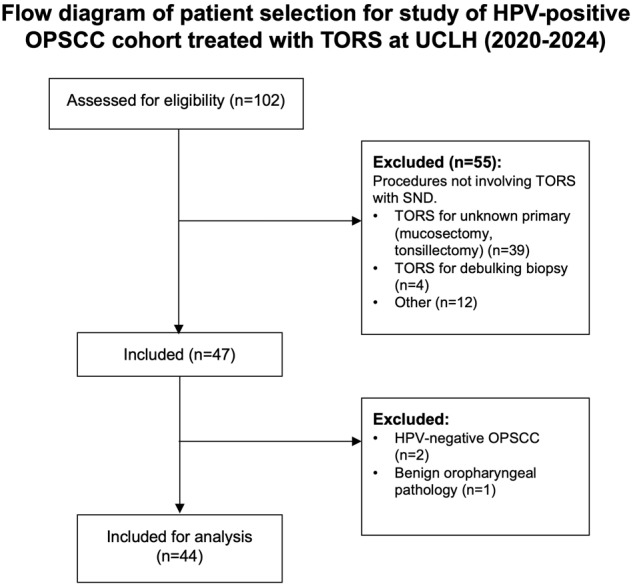
Flow diagram of patient selection for HPV-positive cohort treated with transoral robotic surgery. TORS = transoral robotic surgery; SND = selective neck dissection; HPV = human papillomavirus; OPSCC = oropharyngeal squamous cell carcinoma.

A total of 102 patients were screened, of whom 44 met eligibility criteria. The inclusion criteria were histologically confirmed HPV-positive oropharyngeal SCC (defined by p16 immunohistochemical staining), primary treatment with transoral robotic surgery and selective neck dissection within the study period and a clearly identifiable primary tumour site. The exclusion criterion was diagnostic transoral robotic surgery procedure only (e.g. biopsy, diagnostic tongue base mucosectomy or diagnostic tonsillectomy) (Figure 1).

Of the 44 included patients, 25 were enrolled in the phase III Postoperative Adjuvant Treatment for HPV-positive Tumours trial during the study period.

### Risk stratification and treatment

All patients underwent primary transoral robotic surgery with selective neck dissection of levels II–IV, with inclusion of level I when clinically indicated for anterior tumour extension.^8^

Pre-operatively, patients were assigned an anticipated adjuvant treatment risk category based on clinical tumour–node–metastasis (TNM) staging and radiological findings. This assessment was informed by published Postoperative Adjuvant Treatment for HPV-positive Tumours pathological risk criteria, which were used as a conceptual framework to estimate likely post-operative treatment intensity.

Pathological risk stratification was applied according to the published Postoperative Adjuvant Treatment for HPV-positive Tumours criteria. Low-risk disease (Group A) was defined by the absence of adverse pathological features. Intermediate-risk disease (Group B) included T3 tumours, or T1–T2 tumours with additional risk factors such as N2a/N2b nodal disease, perineural invasion, lymphovascular invasion or close surgical margins (1–5 mm), in the absence of extranodal extension. High-risk disease (Group C) was defined by involved surgical margins (<1 mm) and/or extranodal extension, irrespective of T or N stage.^9^

Post-operatively, this stratification was applied to all patients irrespective of trial enrolment. Patients enrolled in the Postoperative Adjuvant Treatment for HPV-positive Tumours trial received adjuvant therapy according to their allocated trial arm. Patients not enrolled in that trial received standard-of-care adjuvant therapy: no adjuvant therapy for low-risk disease, adjuvant radiotherapy (60 Gy in 30 fractions) for intermediate-risk disease and concurrent cisplatin-based chemoradiotherapy for high-risk disease.

### Data collection

Data were extracted from University College London Hospitals electronic health records, including multidisciplinary team (MDT) documentation and post-operative histopathology reports. For patients receiving adjuvant treatment at North Middlesex University Hospital, supplementary data were extracted from the oncology database.

Collected variables included demographic, pre-operative, pathological and post-operative management factors. Pre-operative clinical variables included Eastern Cooperative Oncology Group performance status,^10^ clinical TNM stage and primary tumour subsite. Primary tumour subsites were grouped as tonsil, base of tongue or other oropharyngeal subsites for analysis.

Pathological variables included pathological TNM stage, primary tumour subsite, surgical margin status, lymph node yield, nodal burden, extranodal extension, lymphovascular invasion and perineural invasion. Surgical margins measuring <1 mm were classified as involved, in accordance with contemporary pathological reporting standards for oropharyngeal SCC, including guidance from the Royal College of Pathologists.^11^ Pathological TNM staging was assigned according to the American Joint Committee on Cancer/Union for International Cancer Control TNM staging system (8th edition) for p16-positive oropharyngeal SCC.^12^ Lymphovascular invasion, perineural invasion and extranodal extension were occasionally reported as ‘suspicious’, ‘possible’ or ‘equivocal’ in histopathology reports. These cases were grouped as indeterminate for analysis to avoid misclassification of adverse pathological features.

Post-operative management variables included the use and modality of adjuvant therapy.

### Data analysis

Descriptive statistics were used to summarise demographic, clinical and pathological characteristics of the study cohort. Transitions between pre-operative clinical nodal staging and post-operative pathological nodal staging were visualised using a Sankey diagram to illustrate patterns of stage concordance and upstaging or downstaging.

### Ethical considerations

This study was conducted in accordance with the ethical principles outlined in the Declaration of Helsinki. Patient confidentiality and privacy were maintained throughout all stages of the research. Approval for the use of anonymised clinical data was obtained through local institutional governance procedures, and individual patient consent was not required.

## Results

Baseline demographic and clinical characteristics are summarised in Table 1. The cohort was predominantly male with Eastern Cooperative Oncology Group/World Health Organization performance status 0.

**Table 1.** Baseline demographic and clinical characteristics of patients with HPV-positive oropharyngeal squamous cell carcinoma treated with primary transoral robotic surgery and selective neck dissection at University College London Hospitals Table 1 long description.

Characteristic	Value
Number of patients	44
Age (median (range); years)	58 (45−73)
Sex (*n* (%))	
– Male	29 (66.0)
– Female	15 (34.0)
ECOG/WHO performance status (*n* (%))	
– 0	42 (95.5)
– 1	2 (4.5)
Primary tumour site (*n* (%))	
– Tonsil	37 (84.1)
– Base of tongue	6 (13.6)
– Other oropharynx	1 (2.3)
Clinical T stage (*n* (%))	
– T1	20 (45.5)
– T2	22 (50.0)
– T3	2 (4.5)
Clinical N stage (*n* (%))	
– N0	7 (15.9)
– N1	34 (77.3)
– N2	2 (4.5)
– N3	1 (2.3)

HPV = human papillomavirus; ECOG = Eastern Cooperative Oncology Group; WHO = World Health Organization

### Primary tumour

Among the 44 patients, tumour subsites were predominantly tonsillar (84.1 per cent, *n* = 37). Most patients had T1–T2 stage disease (95 per cent, *n* = 42), while two patients (4.5 per cent) had T3 disease. Involved margins were identified in 31 patients (70.5 per cent). Two patients returned to the operating theatre for margin clearance; all others were managed with adjuvant therapy.

Lymphovascular invasion was identified in 5 patients (11.4 per cent) and reported as indeterminate in a further 8 patients (18.2 per cent). Perineural invasion was rare, confirmed in 1 patient (2.3 per cent) and reported indeterminate in 2 cases (4.5 per cent). These features contributed to post-operative risk stratification when definitively identified.

### Lymph node assessment

The median lymph node yield was 29 nodes per patient (mean, 33.9 nodes; range, 8–98 nodes). Apart from three cases, all dissections yielded ≥18 lymph nodes. The median number of involved lymph nodes was two (mean, 2.8 nodes, range, 0–15 nodes).

Pathological nodal disease mostly involved level II nodes alone (40.9 per cent, *n* = 18) or in combination with level III disease (20.5 per cent, *n* = 9). Isolated level IIa involvement was observed in 9 patients (20.5 per cent). Six patients (13.6 per cent) had no pathological nodal involvement, while extensive multilevel disease (levels I–IV) was identified in 1 case (2.3 per cent).

Extranodal extension was identified in 14 patients (31.8 per cent) and reported as indeterminate in 2 cases (4.5 per cent).

### Discrepancies between clinical and pathological staging

On pathological assessment, primary tumour stage was upstaged in 6 patients (13.6 per cent) and downstaged in 1 patient (2.3 per cent), with no change observed in the remaining 37 patients (84.1 per cent).

**Figure 2.**
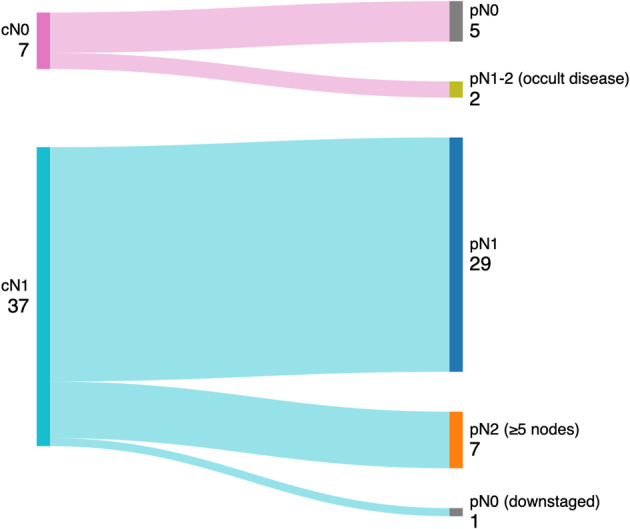
Alluvial (Sankey) diagram demonstrating transitions between clinical nodal stage (cN) and pathological nodal stage (pN), with pathological upstaging to pN2 reflecting five or more metastatic lymph nodes, following transoral robotic surgery and selective neck dissection.

Nodal stage was upstaged in 9 patients (20.5 per cent) and downstaged in a single patient, while 34 patients (77.3 per cent) demonstrated concordant clinical and pathological nodal staging. Of the 9 patients with nodal upstaging, 7 were upstaged from cN1 to pN2 because of identification of a higher-than-anticipated nodal burden (≥5 metastatic lymph nodes). Occult nodal disease resulting in upstaging from cN0 to pN1 or pN2 was observed in two patients Figure 2.

Six of the seven patients upstaged to pN2 also demonstrated extranodal extension on pathological assessment. Post-operative pathology resulted in escalation of adjuvant treatment intensity in 27 patients (61.4 per cent), with 23 patients (52.3 per cent) reclassified from intermediate to high-risk disease according to Postoperative Adjuvant Treatment for HPV-positive Tumours criteria.

At 12 months following surgery, disease-specific survival was 97.7 per cent (*n* = 43). One patient died 11 months post-surgery from metastatic malignancy, with p16-positive liver metastases and a concurrent p16-negative oesophageal SCC.

## Discussion

### Synopsis of key findings

In this consecutive cohort of HPV-positive oropharyngeal SCC patients treated with transoral robotic surgery, post-operative pathological assessment frequently altered risk stratification compared with pre-operative expectations. Involved margins were frequently reported, particularly in tonsillar primaries, and nodal disease was commonly underestimated on pre-operative staging. Identification of extranodal extension on histopathology was a major determinant of post-operative risk reclassification and escalation of adjuvant treatment intensity. Collectively, these findings highlight limitations in pre-operative and intra-operative assessment that may contribute to the escalation of adjuvant treatment in routine clinical practice.

### Strengths

This study provides a comprehensive, real-world evaluation of transoral robotic surgery outcomes in a high-volume UK tertiary centre. All cases were managed within consistent MDT pathways and complete linkage of imaging, operative and histopathological data, minimising missing data. Inclusion of consecutive cases over four years captures institutional practice across an evolving surgical period and provides granular insight into margin status, nodal burden, extranodal extension and staging changes, supporting evaluation of contemporary treatment pathways.

### Comparisons with other studies

The rate of involved margins in this cohort (70.5 per cent) exceeds that reported in other UK (12 per cent)^13^ and North American (4 to 10 per cent) transoral robotic surgery series.^14^^,^^15^ This discrepancy may reflect differences in pathological reporting thresholds, case complexity within a tertiary referral population and evolving surgical practice over the study period. Further work will be required to determine whether refinements in surgical technique or adjunctive technologies, such as robotic flexible fibre carbon dioxide laser systems, can improve margin outcomes in anatomically constrained subsites.

Selective neck dissection involves removal of predefined anatomical nodal levels rather than individual lymph nodes; consequently, lymph node yield is not a direct measure of surgical technical performance. Reported lymph node yield is influenced by patient anatomy and histopathological processing, in addition to the surgical approach. Although lymph node yield varied across the cohort, most dissections met or exceeded commonly used pragmatic adequacy thresholds (≥18 nodes), which have been associated with improved oncologic outcomes and are widely used as a pragmatic surrogate marker of nodal adequacy in head and neck cancer surgery.^16^^,^^17^

The presence of extranodal extension in this study (31.8 per cent) is consistent with published rates of 20–35 per cent in HPV-positive oropharyngeal SCC transoral robotic surgery cohorts.^13^^,^^18^ As demonstrated in previous studies, extranodal extension remains a major determinant of post-operative risk stratification and adjuvant treatment escalation,^19^^,^^20^ despite not being incorporated into TNM (8th edition) pathological nodal staging for HPV-positive disease. This reflects the known limitations of pre-operative imaging in detecting microscopic extranodal extension.^21^^,^^22^

The staging discrepancies observed in this study are also congruent with existing evidence. Clinical tumour staging is generally reliable in HPV-positive oropharyngeal SCC, whereas nodal disease is more frequently underestimated. The nodal upstaging rate of 20.5 per cent closely mirrors that reported in the United States National Cancer Database (20.9 per cent).^23^ In our cohort, nodal upstaging commonly reflected a higher-than-anticipated pathological nodal burden (≥5 metastatic lymph nodes).

A key finding was the high frequency of divergence between anticipated pre-operative and definitive post-operative adjuvant treatment recommendations. Despite MDT pre-operative assessment, post-operative pathology led to escalation of adjuvant treatment intensity in over 60 per cent of patients, with more than half reclassified from intermediate- to high-risk disease according to Postoperative Adjuvant Treatment for HPV-positive Tumours trial criteria. This escalation was driven predominantly by definitive histopathological nodal features, particularly extranodal extension, highlighting the benefit of a surgical-first approach to individualise post-operative therapy.

### Limitations

The study is limited by its retrospective, single-centre design, which may introduce selection bias and limit generalisability. The sample size, while reflective of institutional practice, is smaller than that of multicentre datasets.

Although most histopathological features were reported in a binary manner, a subset of pathology reports used non-definitive descriptors such as ‘suspected’, ‘possible’ or ‘equivocal’ for features including extranodal extension, perineural invasion and lymphovascular invasion. These findings were conservatively classified as indeterminate for the purposes of this analysis, reflecting recognised challenges in pathological interpretation of fragmented or cauterised specimens following transoral resection.

The absence of long-term oncologic, functional and patient-reported outcomes limits assessment of treatment impact beyond immediate pathological endpoints. Given the retrospective and cross-sectional nature of this study, extended follow up and systematic quality-of-life assessment were not feasible. These outcomes are more appropriately addressed by prospective studies, including the Postoperative Adjuvant Treatment for HPV-positive Tumours trial, which is specifically designed to evaluate functional and patient-reported outcomes following risk-adapted treatment in HPV-positive oropharyngeal SCC.

Furthermore, inclusion of Postoperative Adjuvant Treatment for HPV-positive Tumours trial participants introduced heterogeneity in post-operative radiotherapy dose (50 *vs* 60 Gy), which precluded meaningful retrospective comparison of functional outcomes between treatment groups.

### Clinical applicability

Despite these limitations, this retrospective analysis provides critically relevant insights into contemporary transoral robotic surgery pathways for HPV-positive oropharyngeal SCC. The high frequency of involved margins emphasises the importance of meticulous surgical technique and intra-operative margin assessment. As demonstrated in this and other series, pathological features such as extranodal extension remain key drivers of post-operative risk stratification and treatment escalation. While centres await Postoperative Adjuvant Treatment for HPV-positive Tumours trial results, these data offer institution-specific evidence to inform multidisciplinary decision-making and identify opportunities to refine patient selection and treatment planning to minimise unanticipated escalation of adjuvant therapy while preserving function.

- Human papillomavirus-positive oropharyngeal squamous cell carcinoma has a favourable prognosis; consequently, contemporary management focuses on maintaining excellent oncological control while minimising long-term treatment-related morbidity
- Transoral robotic surgery with selective neck dissection enables definitive pathological assessment, forming the basis of risk-adapted adjuvant de-escalation strategies
- This study demonstrates that discrepancies between pre-operative clinical staging and definitive post-operative pathological staging are common, frequently caused by higher-than-anticipated nodal burden and extranodal extension
- These unanticipated adverse pathological features necessitated an escalation in adjuvant treatment intensity in over 60 per cent of patients, highlighting the value of a surgical-first approach to individualise therapy and overcome the limitations of pre-operative staging.

## Data Availability

The data that support the findings of this study are not publicly available because of patient confidentiality constraints but may be available from University College London Hospitals on reasonable request, strictly subject to approval by the University College London Hospitals Information Governance team and the execution of a formal data-sharing agreement.

## Table 1 Long description

The table summarizes baseline demographic and clinical characteristics for 44 patients with HPV-positive oropharyngeal squamous cell carcinoma treated with primary transoral robotic surgery and selective neck dissection. Median age was 58 years, ranging from 45 to 73. Sex distribution was 29 male (66.0 percent) and 15 female (34.0 percent). Performance status was predominantly 0 in 42 patients (95.5 percent), with 2 patients (4.5 percent) at status 1. Primary tumour site was mainly tonsil in 37 patients (84.1 percent), followed by base of tongue in 6 (13.6 percent) and other oropharynx in 1 (2.3 percent). Clinical T stage was mostly T2 in 22 patients (50.0 percent) and T1 in 20 (45.5 percent), with few T3 cases (2 patients, 4.5 percent). Clinical N stage was largely N1 in 34 patients (77.3 percent), with smaller numbers N0 in 7 (15.9 percent), N2 in 2 (4.5 percent), and N3 in 1 (2.3 percent). Percentages may not sum to exactly 100 percent due to rounding and the small sample size.

Navigate back to Table 1.
